# ﻿Morphological and molecular characterization of *Halamphoravantushpaensis* (Bacillariophyceae, Amphipleuraceae), a new diatom species widely dispersed on the shores of the soda Lake Van (Türkiye)

**DOI:** 10.3897/phytokeys.249.133205

**Published:** 2024-11-15

**Authors:** Elif Yılmaz, Romain Gastineau, Cüneyt Nadir Solak, Ewa Górecka, Rosa Trobajo, Monique Turmel, Claude Lemieux, Christian Otis, Andrzej Witkowski, David G. Mann

**Affiliations:** 1 Institute of Marine and Environmental Sciences, University of Szczecin, Mickiewicza 16A, PL70–383 Poland; 2 Department of Biology, Faculty of Science & Art, Dumlupınar University, 43000 Kütahya, Turkiye; 3 Marine and Continental Waters, Institute for Food and Agricultural Research and Technology (IRTA), Crta de Poble Nou Km 5.5, E-43540 La Ràpita, Catalunya, Spain; 4 Département de biochimie, de microbiologie et de bio-Informatique, Institut de Biologie Intégrativeet des Systèmes, Université Laval, Québec, QC, Canada; 5 Plateforme d’Analyse Génomique, Institut de biologie intégrative et des systèmes, Université Laval, Québec, QC, Canada; 6 Royal Botanic Garden Edinburgh, Edinburgh EH3 5LR, Scotland, UK; † Deceased author

**Keywords:** Alkaline lake, group II introns, inverted repeat, mitochondrial genome, multigene phylogeny, nuclear rRNA genes, plastid genome

## Abstract

In this study, we describe *Halamphoravantushpaensis***sp. nov.**, a newly identified diatom species found in the highly alkaline Lake Van in Eastern Turkey (Türkiye). This new species is characterized morphologically by light and scanning electron microscopy, performed on both wild and cultivated samples. Two monoclonal cultures were submitted to a genome-skimming approach, giving access to the complete sequence of their nuclear rRNA cluster of genes, mitochondrial and plastid genomes. Both strains were highly similar from the genomic point of view, with few mutations noted, although in organellar genomes some of them concerned protein coding genes and were non-silent. Also, the group II intron in the mitochondrial *cox1* gene was found to display a relatively high number of polymorphisms. The plastome also distinguishes itself from other *Halamphora* spp. by the extension of its inverted repeat at the expense of the two single copy regions of the genome. Maximum likelihood molecular phylogeny inferred from a concatenated three genes dataset *(18S*, *psbC* and *rbcL*) positions this species within the K clade, which is known to contain hypersaline to freshwater species.

## ﻿Introduction

Soda lakes are among the rarest and most geochemically distinctive wetlands on Earth. They are characterized by their alkaline waters containing high levels of carbonate and bicarbonate ions, typically resulting in elevated pH levels. Lake Van is the largest soda lake in the world (Glombitza et al. 2013), with water that is both saline (21.4‰) and alkaline (155 m mEq^-1^, pH 9.81) (Aydar et al. 2003; Kempe et al. 1991). The lake has existed for 600,000 years, spanning multiple glacial–interglacial cycles (Stockhecke et al. 2014; North et al. 2018) and hosts endemic species of fishes (e.g. Akku et al. 2021). However, studies on the phytoplanktonic flora of the lake, and especially diatoms, have been rather scarce.

For a long time, investigations into the contemporary diatom flora from Lake Van were restricted to a single study by Legler and Krasske (1940). These authors worked on samples brought to them in Germany from Lake Van and, based on light microscopy, they found 24 diatom taxa. Among them, two belonged to the genus *Amphora*. The first one was described as ‘*Amphoracoffeiformis* Ag.’ (more correctly referred to as *Amphoracoffeiformis* (C.Agardh) Kützing, 1844). This is now a non-accepted synonym of *Halamphoracoffeiformis* (C.Agardh) Levkov following the recent revisions of the amphoroid diatoms in which Cleve’s sect. Halamphora has been recognized at the genus level (Cleve 1895; Levkov 2009; Stepanek and Kociolek 2013, 2014, 2019). The second was ‘*Amphoracommutata* Grun.’, published in Van Heurck (1880), considered a valid species as of today. Both taxa are known for being brackish species and both have wide distributions (Guiry and Guiry 2024).

In the last few years, new investigations have been conducted on Lake Van diatoms using an integrative approach that combines light/scanning electron microscopy (LM/SEM) and molecular phylogenies derived from next generation sequencing results. With these data, three new species already have been discovered and described, namely *Nitzschiaanatoliensis* Górecka, Gastineau and Solak (Solak et al. 2021), *Naviculavanseea* Yılmaz, Gastineau, Solak and Witkowski (Yilmaz et al. 2024) and *Halamphorawitkowskii* (Yilmaz et al. in press). Lake Van can be divided into four different basins of various depths: the shallow Erçis basin (northeast), the Van basin (southeast), the Ahlat basin (northwest) and, at the centre of the lake, a deeper fourth basin (Kaden et al. 2010). *Naviculavanseea* and *Ni.anatoliensis* were both described from material from the Erciş basin but have not been observed yet in other parts of the lake. *Halamphorawitkowskii* is known so far only from the Ahlat basin (Yilmaz et al. in press).

In the current article, we describe another *Halamphora* species, *Halamphoravantushpaensis* sp. nov., using the same integrative approach and tools previously used for *Na.vanseea* and *Ni.anatoliensis*.

## ﻿Material and methods

### ﻿Sampling, isolation and cultivation

**Figure 1.**
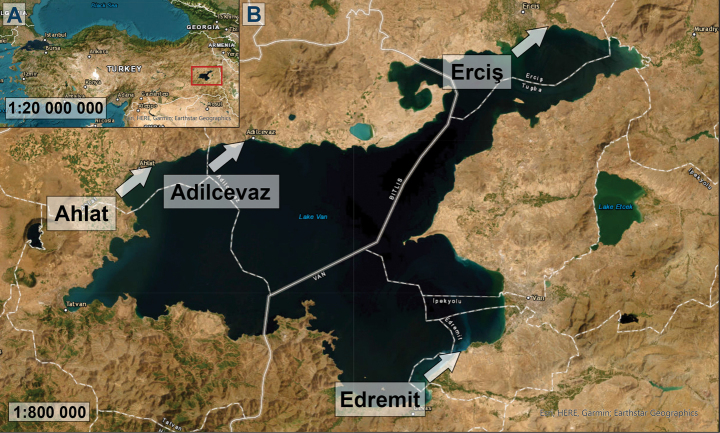
Map of the sampling location **A** location of Lake Van in Turkey. The red frame indicates the position of Lake Van **B** general view of the lake. The areas indicated by arrows are the sampling stations. (Esri. (2023). ArcGIS Pro 3.1.0. Environmental Systems Research Institute)

Live samples were collected by scraping stones at four different stations around Lake Van: Ahlat, Bitlis (38°75'45.748"N, 42°50'71.257"E); Erciş, Van (39°00'07.9"N, 43°25'40.4"E); Adilcevaz, Bitlis (38°79'83"N, 42°72'16"E); and Edremit, Van (38°42'07.09"N, 43°23'74.39"E) (Fig. 1). Individual diatom cells were isolated from the Ahlat samples using a micropipette under a Nikon TS100 inverted microscope (NIKON, Tokyo, Japan). The strains were subsequently moved into 250 mL Erlenmeyer flasks containing F/2 medium (Guillard, 1975), which had been adjusted to 18‰ salinity. The cultures were maintained in conditions promoting active growth, with a light intensity of 60 µmol photons m^-2^ s^-1^, and a photoperiod of 14 hours of light and 10 hours of darkness at a temperature of 18 °C. The two monoclonal cultures were obtained and registered in the Szczecin Diatom Culture Collection as SZCZEY 2166 and SZCZEY 2167.

### ﻿Light and scanning electron microscopy

Light microscopy (LM) documentation was obtained at the University of Szczecin with a Zeiss Axio Scope A1 (Carl Zeiss, Jena, Germany) using a Canon EOS 500D camera and Canon EOS Utility software (Canon, Tokyo, Japan). Images were obtained using a 100× Plan Apochromat oil immersion objective (numerical aperture = 1.4).

For the preparation of diatom frustules for both LM and scanning electron microscope (SEM) observations, samples (pellets of cells from monoclonal cultures or wild samples) were moved into 20 mL beakers and 10 mL of 10% HCl added. Over a 24-hour period, the samples were washed with distilled water four times, allowing the material to sediment naturally between washes. Next, the samples were re-suspended in 30% H_2_O_2_ and boiled on a hotplate at 170 °C for approximately four hours. The final step involved washing the samples four times with distilled water in 24 h, as before. For LM, the material was air-dried on cover glasses and then affixed to a glass slide using Naphrax (Brunel Microscopes, Chippenham, UK). LM measurements were done on a total of 91 valves. For SEM, a drop of cleaned sample was placed on a Nuclepore Track-Etch membrane (Whatman, Maidstone, U.K.). Following air-drying overnight, the membranes were mounted on aluminium stubs using carbon tape and coated with gold using a Q150T coater (Quorum Technologies, Laughton, UK). SEM imaging was conducted at the Faculty of Chemical Technology and Engineering, Western Pomeranian University of Technology in Szczecin (Poland) on a Hitachi SU8020 field emission microscope (Tokyo, Japan). The imaging was conducted with an accelerating voltage of 5kV and a working distance of 8500–8600 µm.

### ﻿Next generation sequencing and bioinformatic analyses

Clones SZCZEY2166 and SZCZEY2167 were harvested by centrifugation and DNA was extracted following Doyle and Doyle (1990). Total DNA was sent to the Beijing genomics Institute (BGI) in Shenzhen (China) where they were sequenced on a DNBSEQ platform for a total for each clone of ca. 40M 150 bp paired-end clean reads. Assemblies were conducted using SPAdes 3.15.0 (Bankevich et al. 2012) with a k-mer parameter of 125. The contigs corresponding to the plastid and mitochondrial genomes or nuclear rRNA clusters were data-mined by standalone blastn queries. The subunits of the plastid genome were merged with each other with the help of Consed (Gordon and Green 2013). Annotation of protein coding genes was done as described in Gastineau et al. (2023).

### ﻿Molecular phylogeny

Maximum likelihood phylogenies were inferred from two different datasets. One contained a concatenated alignment of *18S*, *rbcL* and *psbC* genes representing 214 taxa downloaded from GenBank appended with those obtained in the course of this study. Two strains of *Triparmapacifica* (Guillou and Chrétiennot-Dinet) Ichinomiya and Lopes Santos were used as an outgroup. Among the diatom taxa, two were lacking *rbcL* data, 22 strains lacked *psbC* and 21 strains lacked 18S. A rbcL-only tree was built as well, in order to compare the tree topologies. Sequences were aligned using MAFFT 7 (Katoh and Standley 2013) and trimmed automatically with trimAl (Capella-Gutiérrez et al. 2009). The best model of evolution was selected separately for each gene with ModelTest-NG (Darriba et al. 2020). In case of three-gene dataset, trimmed alignments of *18S*, *rbcL* and *psbC* were concatenated with Phyutility 2.7.1 (Smith and Dunn 2008). Maximum Likelihood phylogenes were computed using IQ-TREE 2.2.0 (Minh et al. 2020) with 1000 ultrafast bootstrap replicates; the dataset was partitioned based on the best models of evolution found for each gene. The trees were visualised with MEGA11 (Tamura et al. 2021). Lists of the sequences with their corresponding accession numbers can be accessed as explained in the data availability statement.

## ﻿Results

### ﻿Taxonomy

**Phylum Bacillariophyta Haeckel**

**Class Bacillariophyceae Haeckel**

**Family Amphipleuraceae Grunow**

**Genus *Halamphora* (Cleve) Levkov**

#### ﻿ Halamphora vantushpaensis

Taxon classificationPlantaeNaviculalesAmphipleuraceae

Yilmaz, Solak & Gastineau sp. nov.

44FB055A-9511-5420-9C91-7A0105130623

Figs 2 , 3 , 4

##### LM

**Figure 2.**
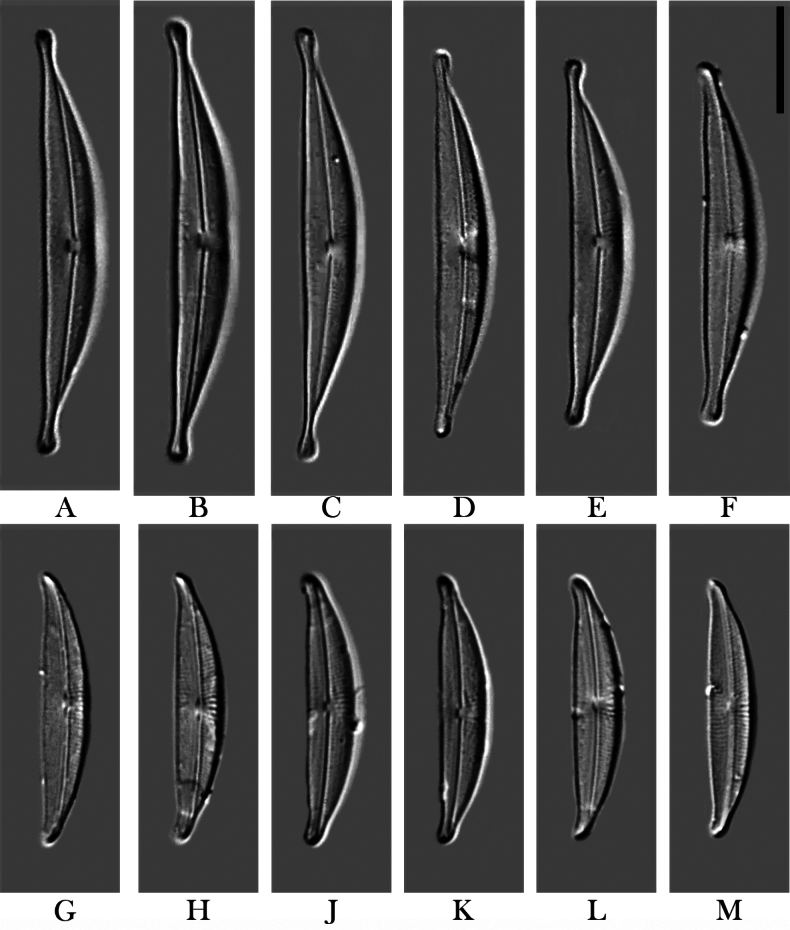
**A–M***Halamphoravantushpaensis* sp. nov. LM micrographs **A–F** cleaned valves of the larger specimen (SZCZEY2167) **G–M** cleaned valves of the smaller specimen (SZCZEY2166). Scale bar: 10 μm

**(Figs 2A–M).** Valves semi-lanceolate, dorsiventral with arched dorsal margin and slightly tumid ventral margin. Valve ends protracted and capitate in larger specimens (Figs 2A–F); but less protracted and not clearly separated from the rest of the valve in smaller specimens (Figs 2G–M), ventrally bent. Valve length 24.0–42.0 µm, valve width 4.0–5.0 µm (n = 35). Axial area very narrow, wider on the ventral side. Central area visible in larger specimens: indistinct on the dorsal side, semi-lanceolate on the ventral side. Raphe almost straight, slightly arched, appearing to be located near the median line of the valve or slightly dorsal in valve view (Fig. 2). Sometimes the proximal raphe endings can be seen to be slightly dorsally bent (Fig. 2B). Striae hard to resolve in LM, dorsally slightly radiate over the entire valve (see SEM images for clearer demonstration of this), 27–32 in 10 µm.

##### SEM

**Figure 3.**
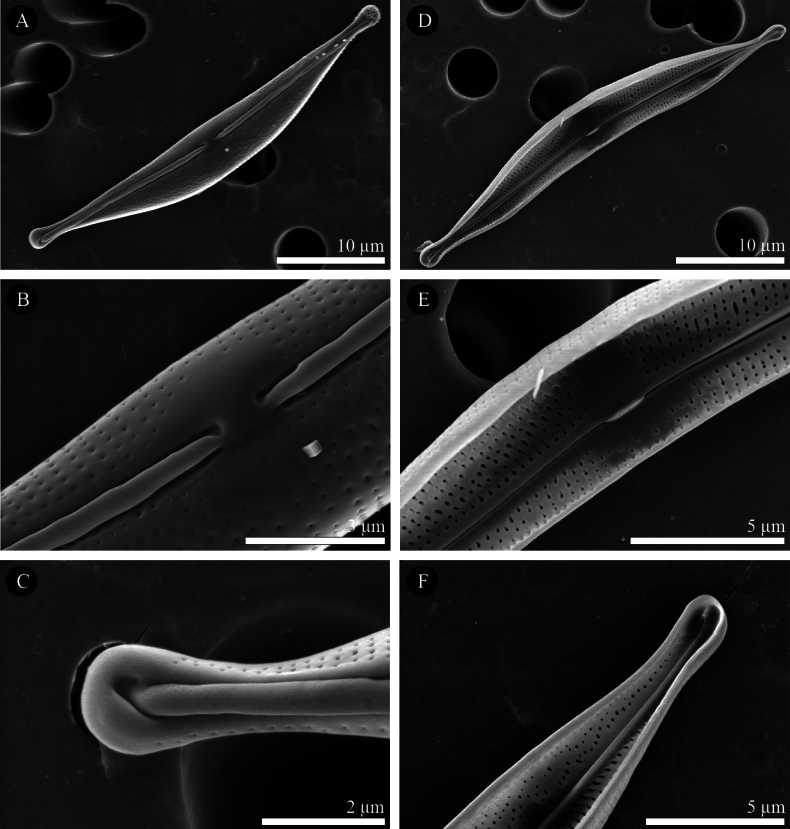
**A–F***Halamphoravantushpaensis* sp. nov. SEM micrographs of strain SZCZEY2167 **A** External view of the entire valve **B** details of central area showing simple proximal raphe endings and regular shortened striae **C** details of apex showing the terminal fissure **D** internal view of the entire valve **E**details of central area showing fused central helictoglossae in proximal raphe endings **F** details of apex showing well-developed helictoglossae. Scale bars: 10 μm (**A, D**); 5 μm (**E, F**); 3 μm (**B**); 2 μm (**C**).

**Figure 4.**
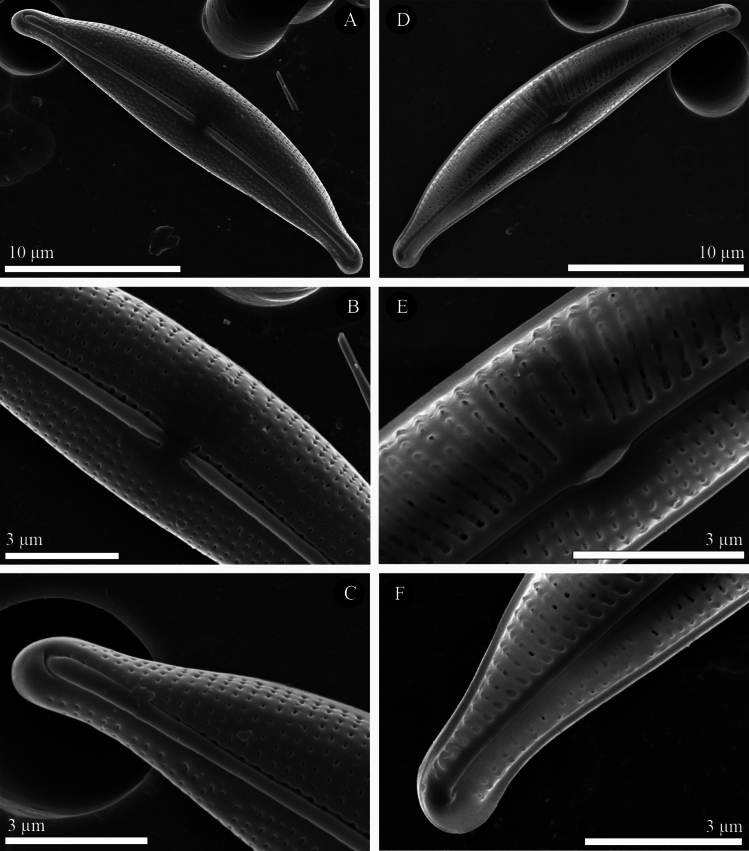
**A–F***Halamphoravantushpaensis* sp. nov. SEM micrographs of strain SZCZEY2166 **A** external view of the entire valve **B** details of central area showing simple proximal raphe endings and regular shortened striae **C** details of apex showing the terminal fissure **D** internal view of the entire valve **E** details of central area showing fused central helictoglossae in proximal raphe endings **F** details of apex showing well-developed helictoglossae. Scale bars: 10 μm (**A, D**); 3 μm (**B, C, E, F**).

**Figure 5.**
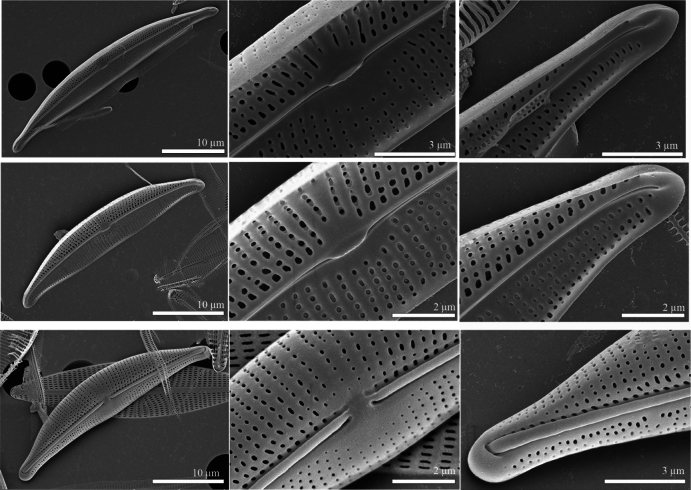
**A–J**SEM images of a cleaned valve from wild material **A** external view of the entire valve of the larger specimens **B** details of central area showing simple proximal raphe endings and regular shortened striae **C** details of apex showing the terminal fissure **D** internal view of the entire valve of the smaller specimens **E** details of central area showing fused central helictoglossae in proximal raphe endings **F** details of apex showing well-developed helictoglossae **G** external view of the entire valve of the smaller specimens **H** details of central area showing simple proximal raphe endings and regular shortened striae **J** details of apex showing the terminal fissure. Scale bars: 10 μm (**A, D, G**); 3 μm (**B, C, J**); 2 μm (**E, H, F**).

**(Figs 3A–F, 4A–F, 5A–J).** Externally, the valve face is arched, merging gently into the mantles (Figs 3B, 4B, C, 5H). Raphe ledge narrow and linear, present on the dorsal side of the raphe, with a prominent groove separating it from the valve face. The proximal raphe endings are slightly expanded into central depressions and are dorsally deflected (Figs 3B, 4B, 5H). The distal raphe endings are dorsally deflected and hook around to link with the groove bordering the raphe ledge (Figs 3C, 4C, 5J). The striae are simple and uniseriate, containing small round or slightly elongate poroids (Figs 3B, 4B, C, 5H), which are somewhat irregularly spaced (Fig. 4B, 5H and see also the internal views in Figs 3E, 4E, 5B, E).

The internal view of the valve shows the overall structure (Figs 3D, 5A). The central area is easier to detect than the external area and appears symmetrical and large on the dorsal side in larger specimens (Fig. 3E, 5B); but very small on both sides in smaller specimens (Fig. 4E, 5E). Proximally, the raphe terminates within a fused central helictoglossa (Figs 3E, 4E, 5E). The distal raphe endings are slightly deflected ventrally and terminate in well-developed helictoglossae (Figs 3F, 4F, 5C, F). Internally, the poroids have round to elliptical internal openings (Figs 3E, 4E, 5B, E). These characteristics are summarized and compared with those of similar species in Table 1.

**Table 1.** Morphological characteristics of *Halamphoravantushpaensis* sp. nov. and similar *Halamphora* (-- represents no information) (for *H.vantushpaensis* measurements, n = 35).

	*Halamphoravantushpaensis* sp. nov.	*H.atacamana*	*H.borealis*	*H.gasseae*	*H.salinicola*	*H.sardiniensis*	*H.thermalis*
Valve length (µm)	**24.0–42.0**	29.0–45.0	19.0–40.0	19.0–35.0	20.0–34.0	13.0–27.5	18.0–40.0
Valve width (µm)	**4.0–5.0**	4.5–8.0	3.0–4.0	3.5–4.5	2.5–3.7	3.0–4.5	4.0–6.5
Stria density (in 10 µm)	**27–32**	25–28	20–24	20–24	21–26	36–42	26–36
Valve outline	**semi-lanceolate with arched dorsal margin, slightly tumid ventral margin**	semi-lanceolate, arched dorsal margin, concave or straight to weakly tumid ventral margin	semi-lanceolate, arched dorsal margin, straight to weakly tumid ventral margin	semi-lanceolate, smoothly arched dorsal margin, straight to weakly concave ventral margin	semi-lanceolate, smoothly arched dorsal margin, straight to weakly concave ventral margin	semi-lanceolate, strongly arched dorsal margin and straight to slightly concave ventral margin	semi-lanceolate to lanceolate, smoothly arched dorsal margin, straight to slightly tumid ventral margin
Valve ending	**subprotracted in smaller specimens; protracted, capitate in larger specimens and ventrally bent**	slightly subprotracted and ventrally bent	protracted, capitate and slightly ventrally bent	shortly protracted and capitate	shortly protracted and capitate	shortly protracted and capitate	attenuate
Raphe ledge	**narrow, arched with equal width throughout**	narrow, equal width throughout and dorsally elevated from the valve face	narrow, linear	--	narrow, expanded on both valve sides	narrow, expanded on both valve sides	narrow, equal width throughout
Axial area	**narrow, widening ventrally**	narrow, widening ventrally	narrow, widening ventrally	narrow, widening ventrally	narrow, widening ventrally	narrow, widening ventrally	narrow, slightly dorsally bent
References	**in this study**	Levkov 2009	Levkov 2009	Levkov 2009	Levkov 2009	Levkov 2009	Levkov 2009

##### Phycobank.

http://phycobank.org/104935.

##### Holotype.

Slides number SZCZEY2167 in the collection of Andrzej Witkowski at the University of Szczecin, Poland. Valves representing the holotype population here illustrated in Fig. 2D.

##### Isotype.

Slide number TR_Erciş_Van_2021 deposited in Kütahya Dumlupınar University (Türkiye).

##### Type locality.

Erciş Van, Turkey (39°00'07.9"N, 43°25'40.4"E) leg. Elif Yılmaz, 31 July 2021.

##### Etymology.

The species is named with regard to both Lake Van and the city of Tushpa, capital of the Iron Age kingdom of Urartu, which was located in the vicinity of the lake.

##### Distribution.

The presence of this taxon has been assessed and confirmed at four different stations around Lake Van: Ahlat (North West of the lake), Adilcevaz (North), Erciş (North East), and Edremit (South East).

### ﻿Genomics - the cluster of nuclear ribosomal RNA genes

Complete clusters of the rRNA genes, containing *18S*, internal transcribed spacer 1 (ITS1), *5.8S*, internal transcribed spacer 2 (ITS2) and *28S*, were obtained for both strains and registered with GenBank accession numbers PP726705 and PP726703 for SZCZEY2166 and SZCZEY2167 respectively. Their sizes and sequences were identical except for a single T/G SNP in the ITS1. The sizes of the different parts of the cluster are indicated in Table 2 and compared with results obtained on the same set of species as in Hamsher et al. (2019), which concern *Halamphoraamericana* Kociolek, 2014, *Halamphoracalidilacuna* Stepanek & Kociolek, 2018 and *H.coffeiformis*. Lengths of *18S*, *5.8S* and *28S* were very conserved among species, except for *H.americana*, which has a group II intron in its *18S* that also contains on ORF coding for a putative reverse transcriptase protein. *Halamphoravantushpaensis* sp. nov. displays a longer ITS1 when compared to other species.

**Table 2.** Length (in bp) of the different portions of the nuclear rRNA cluster for four species of *Halamphora* spp. The length of the *18S* gene of *H.americana* is indicated with and without the intron.

Species	*Halamphoravantushpaensis*	*Halamphoracalidilacuna*	*Halamphoraamericana*	*Halamphoracoffeiformis*
Accession number	PP726705 and PP726703	MH810165	MH810166	MH810167
Total length	5932	5764	9254	5938
18S	1767	1769	1783 (5241 with intron)	1767
ITS1	431	223	229	347
5.8S	156	155	155	154
ITS2	368	405	419	454
28S	3210	3212	3210	3217

### ﻿Genomics - mitochondrial genome

**Figure 6.**
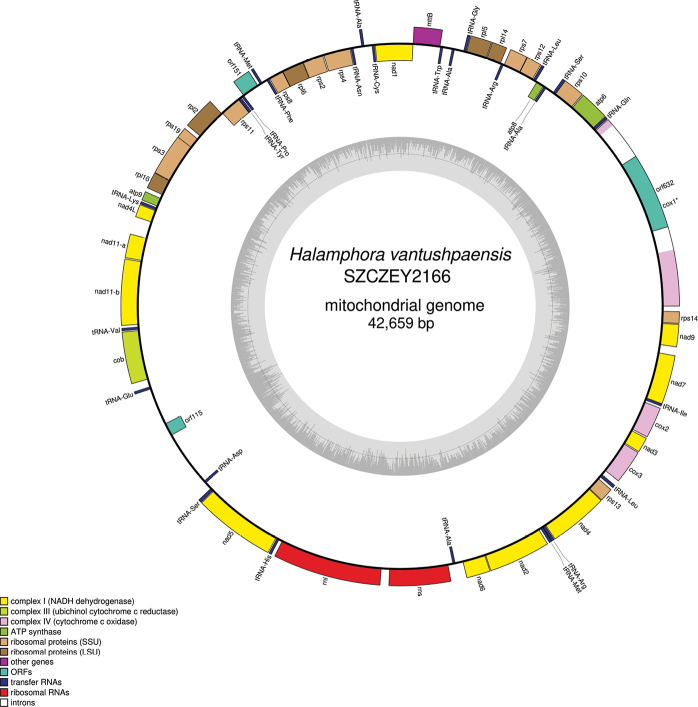
Map of the mitochondrial genome of *Halamphoravantushpaensis* sp. nov. SZCZEY2166

**Figure 7.**
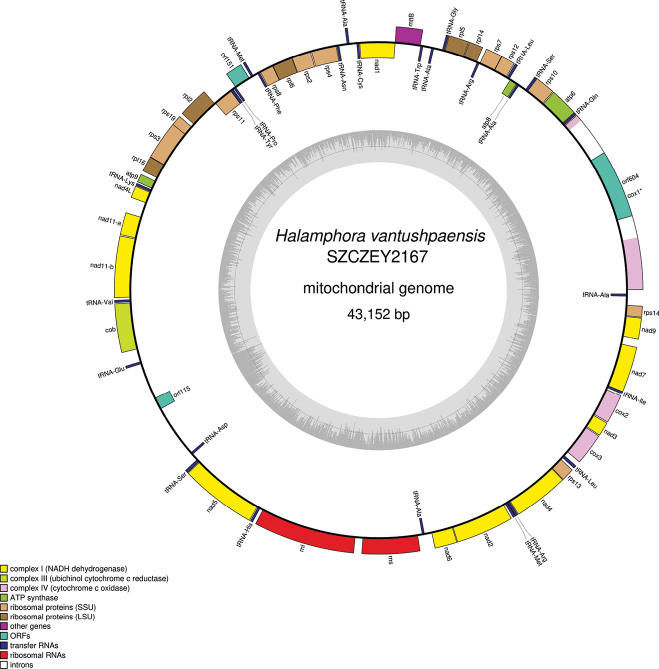
Map of the mitochondrial genome of *Halamphoravantushpaensis* sp. nov. SZCZEY2167

Complete mitogenomes were obtained on both strains and registered with GenBank accession numbers PP962256 (SZCZEY2166) (Fig. 6) and PP962257 (SZCZEY2167) (Fig. 7). The genomes are 42,659 bp and 43,152 bp long (SZCZEY2166 and SZCZEY2167 respectively). The genomes both contain 35 conserved protein coding genes (PCGs), two rRNA and 26 tRNA genes. The mitogenomes harbour the conserved open reading frame (ORF) orf151, although its position differs from most know species among which it is included in a conserved cluster of genes together with *rps11* and *mttb*/*tatC* (Pogoda et al. 2019) whereas here it is located between *rps11* and *rps8*. There is also a non-conserved ORF (orf115) between *cob* and *nad5*. The *cox1* gene contains a group II intron with an ORF coding for a putative reverse transcriptase. There are discrepancies in the length of this putative protein which is 632 amino-acid long in SZCZEY2166 while it is 604 amino-acid long in SZCZEY2167, the extra-length being at the C-terminal part of the putative protein entirely. The polymorphisms between both strains mostly occurred in intergenic parts, hence the slight differences in lengths of the mitogenomes. The conserved protein coding genes were strongly conserved with some of them completely identical, although a certain number of polymorphisms could still be spotted in PCGs in the following genes, with the number of SNPs/lengths indicated between brackets: *cob* (6/1287), *nad2* (1/1536), *nad4* (1/1473), *rpl2* (1/810), *rpl6* (1/573), *rps3* (2/1038), *rps10* (1/540). These mutations were silent in *nad4* and *rpl2*, but led for each of the other encoded protein to one amino-acid substitution. Several variations were otherwise found in the *cox1* intron, whose size varied because of indels (four in total). It otherwise displays 17 SNPs for a total length of 3433/3435 bp, with nine of them being found in the 1815 bp shared between the two putative reverse transcriptase encoding ORF, leading to seven amino-acid substitutions.

### ﻿Genomics - plastid genome

**Figure 8.**
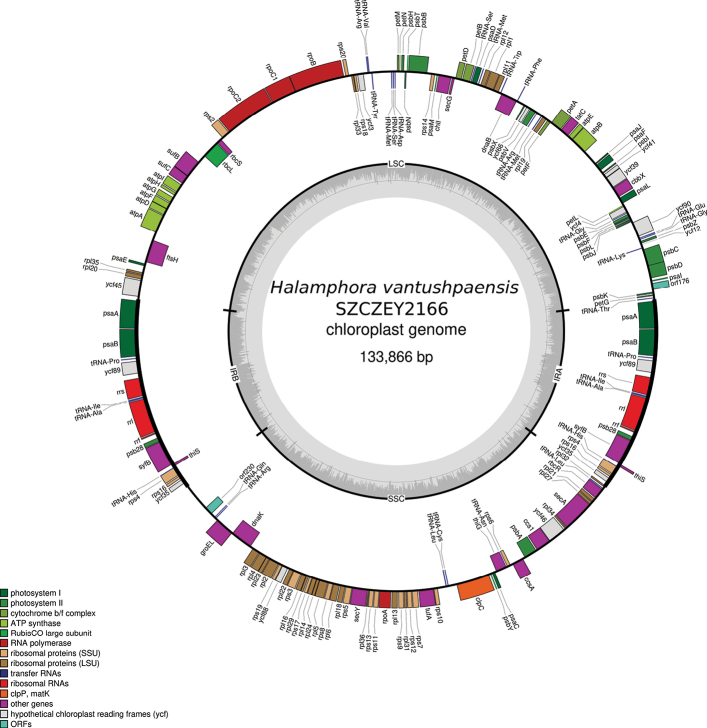
Map of the plastid genome of *Halamphoravantushpaensis* sp. nov. SZCZEY2166

**Figure 9.**
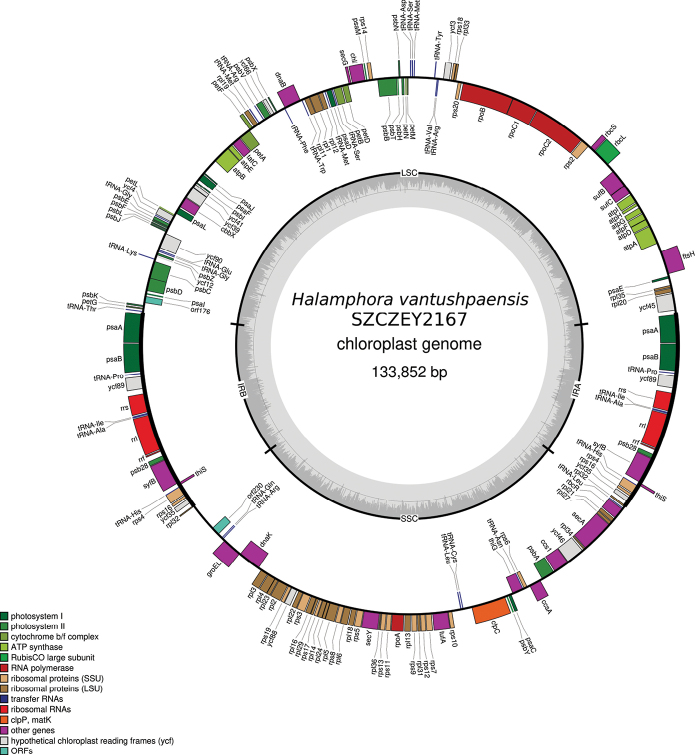
Map of the plastid genome of *Halamphoravantushpaensis* sp. nov. SZCZEY2167

Both plastome were also obtained. Their lengths are 133,866 bp long for strain SZCZEY2166 (GenBank: PP962255) and 133,852 bp long for strain SZCZEY2167 (GenBank: PP727123). The two plastomes came out as different isomers after assembly, hence the difference of strand of the large single copy region (LSC) that can be observed between SZCZEY2166 (Fig. 8) and SZCZEY2167 (Fig. 9). There were slight differences of lengths for LSC and SSC. The LSC is 61,711/61,691 bp long and display 26 indels and six SNPs. Out of these six SNPs, five were found in PCGs (*psbC*, *ycf90*, *petB*, *rps20* and *rpoC2*) and were silent only in *petB* and *rpoC2*. The short single copy (SSC) is 39,615 bp long for both strains, with no indels and 17 SNPs, all located in intergenic area. The inverted repeat had identical lengths and displayed six consecutive polymorphisms in the intergene between *rpl32* and *ycf35*. The LSC contains 70 PCGs, a single non-conserved open reading frame (ORF), and 17 tRNAs. The SSC encodes for 46 PCGs, also a single non-conserved ORF and six tRNAs. The inverted repeat IR contains 10 PCGs, three rRNA and four tRNA.

Three plastid genomes are available in GenBank for the genus *Halamphora*, all originating from the same study (Hamsher et al. 2019). In Table 3, the total lengths of these genomes and the lengths of their different compartments are compared.

**Table 3.** Lengths (in bp) of the different compartments of the plastid genomes of four species of *Halamphora* spp.

Species	Length of the LSC	Length of the SSC	Length of the IR	Total length
*Halamphoracalidilacuna*	82,227	49,698	9,407	150,739
*Halamphoraamericana*	77,289	44,724	10,269	142,551
*Halamphoracoffeiformis*	64,938	41,485	7,752	121,927
*Halamphoravantushpaensis*	61,705/61,691	39,615/39,615	16,273	133,866/133,852

*Halamphoravantushpaensis* has shorter LSC and SSC but its IR is consequently longer when compared to other species. The gene content of the IR is compared for all these species in Table 4. The restricted set of conserved genes found among *H.calidilacuna* or *H.americana* and which consists of a single PCG (*ycf89*), three tRNA and three rRNA seems to be shared by many unrelated species and genera such as *Naviculaveneta*Kützing 1844 or *Tryblionellaapiculata* Gregory 1857 (Gastineau et al. 2021a). As with *H.americana*, an extension of the IR may result from the presence of non-conserved ORF or putative genes of plasmid origin, as exemplified by its ORF9 and the putative integrase/recombinase encoded by the gene labelled as *tyrC* by Hamsher et al. (2019). The case of *H.vantushpaensis* is entirely different in the sense that the extension of the IR is a consequence of the incorporation of several conserved PCGs plus one tRNA. When compared with the gene content of the other species, it appears that this extension has been done at the expense of both the LSC and the SSC, which distinguishes it from species like *Climaconeis* spp. (Gastineau et al. 2021b) among which the IR seemed to have only taken over the SSC. Indeed, among the other *Halamphora* spp., *psaA* and *psaB* are located in the LSC while the other genes belong to the SSC in which they form a well-conserved cluster.

**Table 4.** Gene composition of the inverted repeats of the plastid genomes of four species of *Halamphora* spp. Genes highlighted in bold for *Halamphoravantushpaensis* sp. nov. are genes found in the LSC in other species. Genes in bold italic concern genes usually found in the SSC. Genes marked by an asterisk are non-conserved genes of probable plasmidic origin.

Species	Gene composition of the IR
*Halamphoracalidilacuna*	*tRNA-Pro, ycf89, rrs*, *tRNA*-*Ile*, *tRNA*-*Ala*, *rrl*, *rrf*
*Halamphoraamericana*	*tRNA*-*Pro*, *ycf89*, ORF9*, *rrs*, *tRNA*-*Ile*, *tRNA*-*Ala*, *rrl*, *rrf*, tyrC*
*Halamphoracoffeiformis*	*tRNA*-*Pro*, *ycf89*, *rrs*, *tRNA*-*Ile*, *tRNA*-*Ala*, *rrl*, *rrf*, *ycf35*
*Halamphoravantushpaensis*	**psaA, psaB**, *tRNA*-*Pro*, *ycf89*, *rrs*, *tRNA*-*Ile*, *tRNA*-*Ala*, *rrl*, *rrf*, ***psb28, syfb, thiS, tRNA-His, rps4, rps16, ycf35, rpl32***

### ﻿Molecular maximum likelihood phylogeny

**Figure 10.**
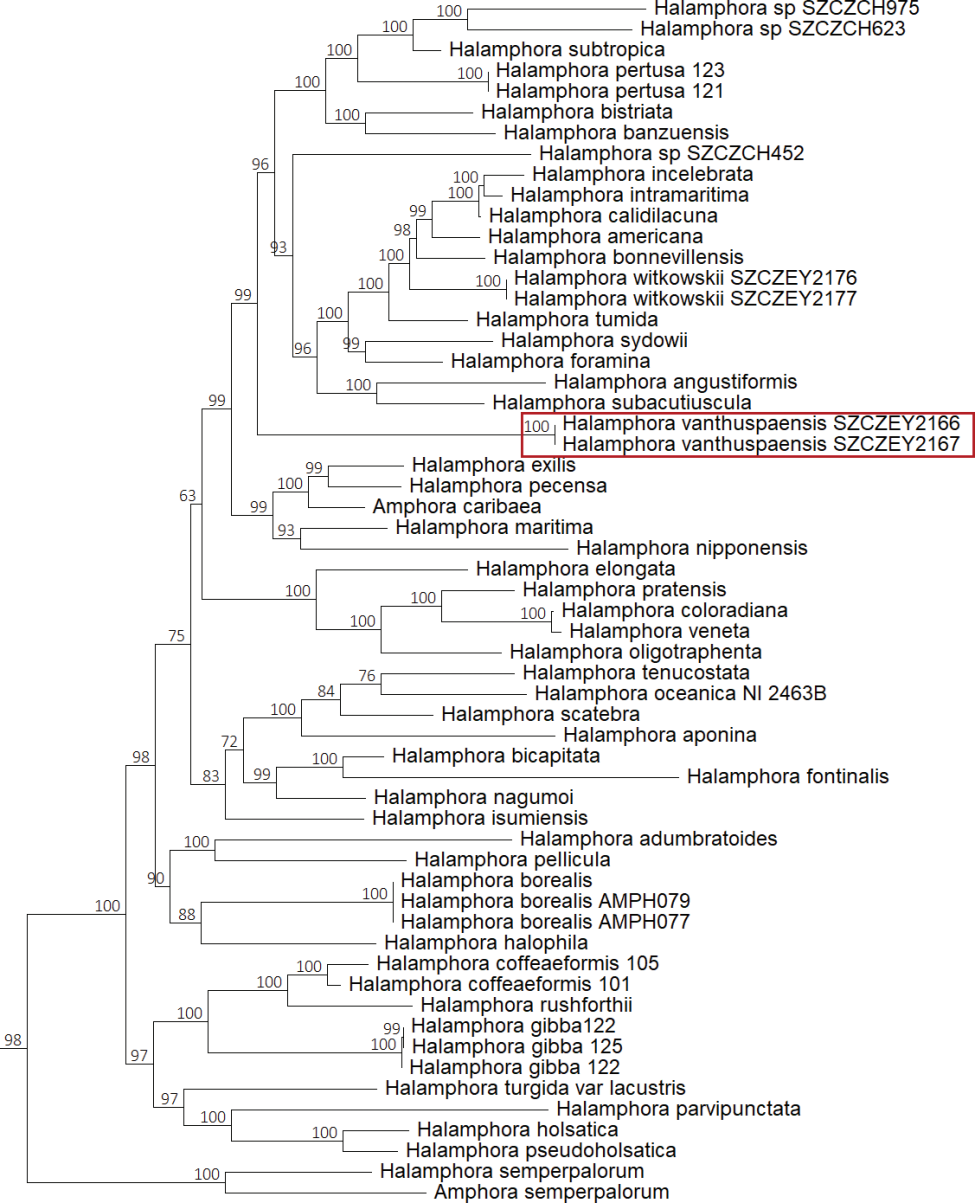
Maximum Likelihood phylogenetic tree inferred from concatenated alignments of *psbC*, *rbcL* and *18S*. The figure represents the sub-tree that contains the *Halamphora* clade.

Fig. 10 presents the *Halamphora* clade as a sub-tree derived from the three-gene inferred phylogeny. The complete three-gene tree and the *rbcL*-only tree can be found as indicated in the data availability statement. In the three-gene tree, *H.vantushpaensis* strains appear as a highly supported (99%) long-branched sister group to a larger cluster composed of 18 *Halamphora* species, namely *H.subacutiuscula*, *H.angustiformis*, *H.foramina*, *H.sydowii*, *H.tumida*, *H.witkowskii*, *H.bonnewillensis*, *H.americana*, *H.calidilacuna*, *H.intramaritima*, *H.incelebrata*, *H.banzuensis*, *H.bistriata*, *H.pertusa*, *H.subtropica* plus three unidentified *Halamphora* species. The topology of the *rbcL*-inferred tree slightly differs regarding the species sister to *H.vantushpaensis*, which are, in this case, *H.angustiformis* (bv = 93) and *H.subacutiuscula* (bv = 96). These strains are further nested in a clade with *H.maritima*, *H.pecensa*, “*Amphora*” *caribeana*, *H.exilis*, *H.subtropica*, *H.pertusa*, *H.banzuensis and H.bistriata* with low support (bv < 50) and, together with these, sister to *H.tumida*, *H.witkowskii*, *H.bonnewillensis*, *H.americana*, *H.calidilacuna*, *H.intramaritima*, *H.incelebrata*, *H.foramina*, *H.sydowii* and *Halamphora* sp. SZCZCH45

## ﻿Discussion

### ﻿Morphological comparison with similar taxa

*Halamphoravantushpaensis* sp. nov. is a new species, characterized through the extensive study of two distinct cultivated clones as well as examination of wild samples. The findings indicate that the morphological characteristics of *H.vantushpaensis* can strongly vary and that LM observations might not be sufficient. *Halamphoraatacamana* (Patrick) Levkov, *H.borealis* (Kützing) Levkov, *H.gasseae* Levkov, and *H.salinicola* Levkov and Diaz have been identified as the most similar species. In terms of outline, *H.borealis* exhibits a semi-lanceolate shape similar to *H.vantushpaensis* (Table 1). However, distinguishing features of *H.vantushpaensis* such as the larger ventral side and the indistinct striae with a higher density (more than 27 striae in 10 µm) set it apart. Additionally, SEM images reveal differences in striae composition between *H.vantushpaensis* and *H.borealis*, further supporting their taxonomic differentiation. *Halamphoraatacamana* exhibits a similar outline, especially to smaller specimens of *H.vantushpaensis*, with slightly protracted valve endings; however, larger specimens of *H.vantushpaensis* have elongated valve ends. *Halamphoraatacamana* also tends to have lower stria densities (< 28 in 10 µm). *Halamphoragassеae* and *H.salinicola* are further similar taxa, but both have smaller valves (< 35 µm in length, < 4.5 µm in width), lower stria densities (< 27 in 10 µm), and smoothly arched dorsal margins. Moreover, we observed that *H.salinicola* has larger areolae on the dorsal side, one elongate areolae on the ventral side, and a raphe ledge that extends continuously over the entire length of the valve. Among other species that might be compared with *H.vantushpaensis*, *H.sardiniensis* has smaller valves and a higher stria density (> 36 striae in 10 µm) and strongly arched dorsal margin. Additionally, *H.sardiniensis* has elongate areolae on the dorsal side, one elongate areola on the ventral side, and a large central area on the ventral side (visible in SEM: Levkov 2009, pl.245, fig. 4). *Halamphorathermalis* is similar to smaller specimens of *H.vantushpaensis*. However, *H.thermalis* has a smoothly arched dorsal margin and a more visibly tumid ventral margin. In SEM, *H.thermalis* has larger irregularly rounded elongate areolae on dorsal side and rounded areolae on ventral side and areolae become smaller toward the central area ventrally. Also, the proximal raphe endings open into larger depressions (Levkov 2009, pl. 230, figs 1–6).

### ﻿Genomic results and phylogenies

Initially, when comparing SZCZEY2166 and SZCZEY2167 by the means of LM, it was unclear whether or not they belonged to identical or different species, particularly because of the differences of shape of their apices. Of course, SEM brought supplementary clues of their identity, but in the end, molecular methods provided the decisive argument. With regards to this, it should be noted that within a single round of short-reads sequencing, complete nuclear rRNA clusters, mitochondrial genomes and plastid genomes were obtained, which allowed to derive accurate phylogenies. The *rbcL*-inferred phylogeny strictly positions *H.vantushpaensis* within a clade of species previously described as ‘K clade’ (Stepanek and Kociolek 2019). This is a noteworthy result, because this clade is known to contain species with extremely different preferences for what regards salinity, ranging from freshwater to hypersaline. As such, this clade is regarded as an interesting model to study transition between habitats, although it is noteworthy that such a transition seemed to have occurred repeatedly and independently among the genus Halamphora. The genomic approach we employed, sometimes described as ‘genome-skimming’, has reliably provided results on diatoms (for recent examples, see Gastineau et al. 2021c; Solak et al. 2021 ; Dąbek et al. 2022 ; Yilmaz et al. 2024). Aside from their interest in molecular phylogeny, the availability of full-length RNA operon reference sequences from duly identified organisms could become increasingly valuable with the development of long-read metabarcoding (e.g. Jamy et al. 2020). In the current case, it was interesting to see that the only polymorphism between both strains of *H.vantushpaensis* was located in one of the internal transcribed spacers, a portion that does not participate to the final 3D structure of the ribosome and as such, is more likely to display variations.

When comparing the plastomes, the low number of polymorphisms slightly misled us at first into thinking that these SNPs might only have concerned non-coding parts. Surprisingly, it was not the case, and although the number of SNPs is rather low, interestingly, several among them were not silent. However, this variability between the two strains is consequently lower than what was observed with the previously published Lake Van-species *N.vanseea* (Yılmaz et al. 2024). A gene such as *psbC* displayed three times more polymorphisms between the two strains studied at that time when compared to what was unveiled between both strains of *H.vantushpaensis*. At the time *N.vanseea* was being investigated, it was possible to sequence the mitogenome for only one of the strains, for reasons that remain unknown but might be related to the amount of bacterial contamination in the DNA pool. This time, with *H.vantushpaensis*, sequencing of both strains was successful. We note that the *cox1* gene, which seems to be a sensitive marker for the study of diatoms at the subspecific level (Gastineau et al. 2013, 2021c; Dąbek et al. 2022) was entirely conserved in its exonic parts. The position of the *cox1* intron was perfectly conserved, unlike previous reports on other species (Gastineau et al. 2021c), but there were noticeable differences in its sequence. This is exemplified by the changes in length and sequence of the putative reverse-transcriptase it contains. Among land plants, introns in organellar genomes have been documented to be relevant as population markers (e.g. Spaniolas et al. 2010; Grosser et al. 2023), but no such work seems to exist for diatoms.

## Supplementary Material

XML Treatment for Halamphora vantushpaensis

## ﻿Additional information

### Author ORCIDs

Elif Yılmaz https://orcid.org/0000-0001-7939-1814

Romain Gastineau https://orcid.org/0000-0001-8661-5118

Cüneyt Nadir Solak https://orcid.org/0000-0003-2334-4271

Ewa Górecka https://orcid.org/0000-0003-0590-7480

Rosa Trobajo https://orcid.org/0000-0001-9498-3797

Monique Turmel https://orcid.org/0000-0001-7060-035X

Claude Lemieux https://orcid.org/0000-0001-9580-8042

Christian Otis https://orcid.org/0000-0001-9680-5863

Andrzej Witkowski https://orcid.org/0000-0003-1714-218X

David G. Mann https://orcid.org/0000-0003-0522-6802

### Data availability

All sequences have been deposited and are already available on GenBank with accession numbers PP726705, PP726703, PP962256, PP962257, PP962255, PP727123. The complete 3-gene tree, the rbcL-inferred trees and the lists of sequences used for phylogeny can be found on Zenodo following this link: https://doi.org/10.5281/zenodo.12963401.
